# Silica-Based Nanomaterials for Diabetes Mellitus Treatment

**DOI:** 10.3390/bioengineering10010040

**Published:** 2022-12-29

**Authors:** Diogo Marinheiro, Fátima Martel, Bárbara J. M. L. Ferreira, Ana L. Daniel-da-Silva

**Affiliations:** 1 Department of Chemistry & CICECO-Aveiro Institute of Materials, University of Aveiro, 3810-193 Aveiro, Portugal; 2 Biochemistry Unit, Biomedicine Department, Faculty of Medicine, University of Porto, 4200-319 Porto, Portugal; 3 I3S—Institute of Research and innovation in Health, University of Porto, 4200-135 Porto, Portugal

**Keywords:** silica nanoparticles, drug delivery, diabetes mellitus, insulin, antidiabetic drugs, hyperglycaemia

## Abstract

Diabetes mellitus, a chronic metabolic disease with an alarming global prevalence, is associated with several serious health threats, including cardiovascular diseases. Current diabetes treatments have several limitations and disadvantages, creating the need for new effective formulations to combat this disease and its associated complications. This motivated the development of therapeutic strategies to overcome some of these limitations, such as low therapeutic drug bioavailability or poor compliance of patients with current therapeutic methodologies. Taking advantage of silica nanoparticle characteristics such as tuneable particle and pore size, surface chemistry and biocompatibility, silica-based nanocarriers have been developed with the potential to treat diabetes and regulate blood glucose concentration. This review discusses the main topics in the field, such as oral administration of insulin, glucose-responsive devices and innovative administration routes.

## 1. Introduction

In 2016, the World Health Organization reported 422 million adults living with diabetes (around 8.5% of the adult population), representing almost four times more individuals than in 1980. This was mainly attributed to type 2 diabetes and its risk factors, including overweight and obesity. Every year, around 1.5 million deaths are directly attributed to diabetes, being the ninth cause of death globally [1]. In Portugal, numbers from 2014 reveal that 9.2% of the population suffered from diabetes, accounting for 5% of deaths at all ages [2]. The increasing prevalence and the severe health risks associated with diabetes triggered the research to develop new therapeutic approaches to fight the increasing incidence of this disease as well as ameliorate the symptoms, life quality and life expectancy of diabetic patients. 

In recent years, new therapeutic strategies have been proposed for diabetes, either for oral delivery of antidiabetic drugs or to achieve a more controlled hypoglycaemic effect. Several nanomaterials of distinct composition, such as lipid [3,4,5,6] and polymer [7,8,9,10] nanoparticles, metal organic frameworks [11,12] and silica nanoparticles [13,14,15], have been reported for improving oral bioavailability of antidiabetic drugs. Lipid nanoparticles increased the oral bioavailability of antidiabetic peptide drugs. However, the clinical application of the system is limited by its low encapsulation efficiency and poor stability in aqueous fluids [16]. Polymer nanoparticles provide good encapsulation efficiency and varied chemistry and can be prepared from biocompatible polymers, such as polylactic acid (PLA) and polyethylene glycol (PEG) [17]. However, they easily disaggregate in biological fluids, which limits the oral bioavailability of the drug and may cause hypoglycaemia if a burst release in the bloodstream happens [16]. Metal organic frameworks have also been studied for diabetes treatment, but to a much lower extent and consequently with many questions still to be addressed, such as stability, intestinal permeation and safety [16]. In contrast, silica nanoparticles have excellent physicochemical stability, good biocompatibility, high loading capacity (porous silica) with controllable particle size, surface area and pore volume, and an easily modifiable surface [16,18]. Accordingly, several studies demonstrate that the capacity of silica nanoparticles to be used has a potential alternative for diabetes therapy [19]. Furthermore, among inorganic coating materials, amorphous silica (SiO_2_) is by far the most frequently used material for bioapplications [20]. The silica shell provides a springboard for direct functionalization with readily available organofunctional silane coupling agents for subsequent conjugation with drugs or biomolecules [21,22,23].

Mesoporous silica nanoparticles (MSNs) are the most frequently studied in the context of antidiabetic therapy, but other silica nanocarriers have also been reported [24]. Using MSNs, several molecules with antidiabetic effects have been investigated, with a particular focus on insulin [13,14,15]. Although the antidiabetic effects of various polyphenols are well known [25,26] nanosilica-loaded polyphenols for diabetes treatment have not yet been reported [27]. Nevertheless, these systems have been tested in the treatment of other diseases, including different types of cancer such as breast cancer [28,29], prostate cancer [30], melanoma [31] and others [32], as well as cardiovascular diseases [33].

Here, we present a review of the work that has been developed employing silica-based nanoparticles for treating diabetes. The main approach of the works described here aims to control/reduce glucose blood levels, yet other therapeutic goals have also been proposed. First, we introduce the diabetes mellitus disorder and the existing treatments, followed by a brief description of silica nanoparticle properties and synthesis methods, emphasizing MSNs. Then, we discuss the nanosystems proposed for insulin delivery through different administration routes, their characteristics, strengths, and limitations, as well as for other drugs with hypoglycaemic effects. The main objective of this review is to compile relevant works and provide a critical point of view on silica nanoparticles’ usage in managing diabetes and its related complications.

## 2. Diabetes Mellitus

**Figure 1:**
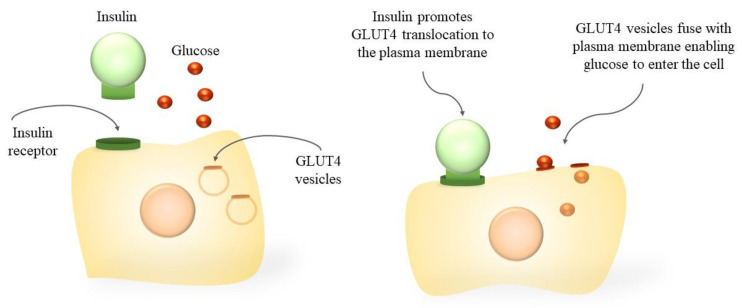
Representation of the mechanism of action of insulin on the cellular uptake of glucose through GLUT4.

Diabetes mellitus, commonly known as diabetes, is a set of metabolic disorders characterized by hyperglycaemia related to a deficiency in insulin secretion, insulin action or both [34]. Insulin is a peptide hormone synthesized by the β-cells of the pancreas, responsible for regulating the storage and release of energy during feeding and fasting, controlling blood glucose levels [35]. Among its many effects, this peptide stimulates GLUT4-mediated cellular glucose uptake in muscle and adipose cells. The transport respects the gradient concentration through facilitated diffusion, not requiring ATP. Insulin receptors recognize insulin and allow glucose to be transported through the cellular membrane (Figure 1) [36]. The symptoms of diabetes may include frequent urination, increased hunger and thirst, weight loss, and unconsciousness [37].

Although there are many subclassifications of diabetes, its two main forms are type 1 and type 2 diabetes. Other forms of diabetes are less common and include gestational diabetes, which develops during pregnancy. Furthermore, any diseases of the exocrine pancreas that damage the pancreas can result in diabetes, including pancreatic cancer and cystic fibrosis, drugs or chemicals, and infections such as congenital rubella. Several genetic disorders lead to the development of diabetes, such as maturity-onset diabetes of the young, caused by genetic defects of β-cell function, genetic defects in insulin action genetic syndromes, including Down syndrome and Klinefelter syndrome, among many others [34,38]. 

Type 1 diabetes is autoimmune-mediated diabetes caused by the destruction of the β-cells of the pancreas by the immune system, usually leading to absolute insulin deficiency. Only 5 to 10% of diabetic individuals have this type of diabetes [34].

Type 2 diabetes is mainly caused by insulin resistance in target cells, leading to alterations in β-cells size and function. Patients often suffer from both defects in insulin secretion and action, making it unclear which anomaly is the primary cause of hyperglycaemia [34]. Due to a compensation mechanism of the body attempting to produce more insulin, the body increases insulin production to combat hyperglycaemia, leading to an increase in the size of islet cells and pancreatic β-cells. On the other hand, chronic hyperglycaemia caused by chronic over-nutrition has proven to induce β-cells apoptosis, diminishing their function. Apoptosis is caused by endoplasmic reticulum stress, a high intracellular calcium level, the production of reactive oxygen species (ROS) and oxidation stress [37]. This type of diabetes is the most common, with approximately 90 to 95% of total cases. This form of diabetes usually occurs in adults with insulin resistance and presenting relative insulin deficiency. Nevertheless, these individuals normally do not need insulin treatment to survive [34]. There are probably many different triggers for this form of diabetes. However, most individuals with type 2 diabetes are obese, and even those not obese according to standard weight criteria may have a higher percentage of body fat distributed mainly in the abdominal area [34,39].

### 2.1. Health Risks

The persistent hyperglycaemia caused by diabetes is linked to long-term damage, malfunction and failure of several organs, including the eyes, kidneys, nerves, heart and blood vessels [34]. Diabetes is a well-known risk factor for coronary heart disease and ischemic stroke. By itself, diabetes increases from two to three times the risk of coronary heart disease, major stroke subtypes, and deaths attributed to other vascular causes. Diabetic people tend to have more severe forms of coronary lesions. In fact, diabetes is three times more strongly related to fatal myocardial infarction than to non-fatal myocardial infarction. Actually, 10% of vascular deaths in adults in developed countries have been attributed to diabetes [40].

Other severe health problems might be caused by diabetes. Around 1 million people are blind due to diabetes. Diabetic retinopathy is the leading cause of blindness, and it is caused by long-term damage to the small blood vessels in the retina [41]. Diabetes is also among the leading causes of kidney failure [42].

### 2.2. Treatments 

An efficient diabetes treatment must guarantee optimum and balanced blood glucose concentrations, as well as reduce long-term diabetes-related complications [34].

Since type 1 diabetes is caused by a lack of insulin production and secretion, insulin administration is the crux of treatment. However, insulin therapy alone fails to achieve target glycaemic control in the majority of individuals with type 1 diabetes and is associated with side effects, such as hypoglycaemia and weight gain [43]. For type 2 diabetes, diet and exercise may be sufficient therapies, but if these lifestyle changes do not yield adequate improvement, then drug treatment should be initiated. In some cases, insulin treatment may also be required, particularly in patients with abnormal glucose control in the latter phase of the disease. The major non-insulin-based oral therapies for type 2 diabetes include sulfonylureas and meglitinides (insulin secretagogues), biguanides (reduce hepatic glucose production and glucose intestinal absorption), peroxisome proliferator-activated receptor-γ (PPARγ) agonists (thiazolidinediones/glitazones) (insulin sensitizers), GLP-1 agonists and dipeptidyl peptidase-IV inhibitors (incretin mimetics), α-glucosidase inhibitors (e.g., acarbose) (reduce the intestinal absorption of glucose), amylin analogues (pramlintide acetate) (delay gastric emptying, suppress the secretion of glucagon and inhibit appetite) and SGLT2 inhibitors (gliflozins) (reduce the renal reabsorption of glucose) [44]. Although oral hypoglycaemic drugs (either administered as monotherapy or given in combination) are generally considered safe, as with many other drugs, they are associated with unwanted side effects, such as severe hypoglycaemia, weight gain, gastrointestinal symptoms such as metallic taste in the mouth, abdominal distress and diarrhoea, mild anorexia and nausea [44,45,46]. Moreover, lower therapeutic efficacy owing to improper or ineffective dosage regimen, altered side effects due to drug metabolism and lack of target specificity, solubility and permeability problems are major drawbacks associated with the use of the above-mentioned conventional drugs [44].

Therefore, circumventing the stated issues related to conventional drug usage is necessary [39]. In this context, nanoformulations present numerous benefits and have emerged as an interesting approach. Nanoparticles not only increase the solubility of the drug but also allow for a reduction in the dosage, a rapid onset of action, a controlled drug release profile, fewer side effects, optimized drug delivery, expanded drug half-life, minimized patient variability, and optimized bioavailability. So, they can resolve several of the drawbacks of current antidiabetics [47,48]. Moreover, nanoformulations often promote cellular drug uptake or disrupt cellular efflux mechanisms, such as the P-glycoprotein or target particular receptors that further strengthen the pharmacokinetics and pharmacodynamics profile of numerous antidiabetic molecules [44].

## 3. Silica Nanoparticles

### 3.1. Types and Properties

Nanotechnology is the science of engineering materials and systems on a scale usually less than 100 nanometres [49]. It involves developing diverse organic and inorganic-based nanomaterials, using various components, including phospholipids, polymers such as chitosan, dextran and polyethylene glycol (PEG), cholesterol, carbon, silica and several metals [50]. Nanoparticles are similar in size to many vital biomolecules, such as antibodies, membrane receptors, nucleic acids, and proteins. These mimicking size features, together with their high surface area to volume ratio, make nanoparticles a powerful tool in modern nanomedicine [51].

**Figure 2:**
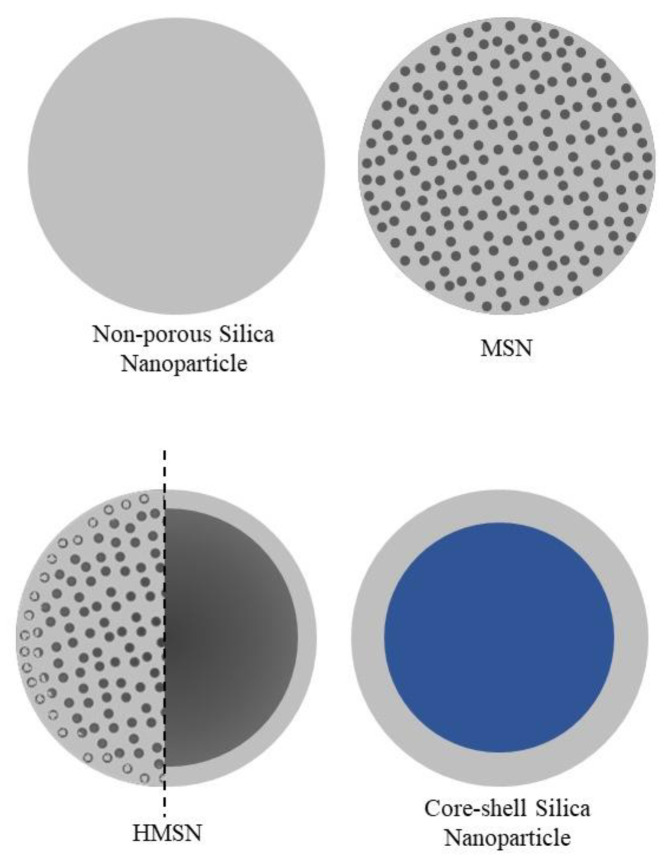
Types of silica nanoparticles: non-porous silica nanoparticles, mesoporous silica nanoparticles (MSN), hollow mesoporous silica nanoparticles (HMSN) and core-shell silica nanoparticles. The grey colour represents silica materials, while blue represents the nanoparticle’s core, which is a different material.

Silica nanoparticles are inorganic nanoparticles of different types, including the ones represented in Figure 2: conventional non-porous silica nanoparticles, MSNs, hollow mesoporous silica nanoparticles (HMSN) and core-shell silica [18]. MSNs and HMSNs are the most commonly employed silica-based nanoparticle treatments for diabetes [52,53,54], and for this reason, the ones that will be more detailed addressed in this review. Silica nanoparticle applications have been widely investigated in the realm of biomedicine, namely as nanocarriers to load a wide range of cargo, from medicines to macromolecules such proteins, DNA and RNA [55]. 

Mesoporous silica-based materials were first reported in the early 1990s by Mobil Company [56]. They are highly attractive for drug delivery due to their well-defined and controllable microstructure and excellent biocompatibility [57]. The superior chemical, mechanical and textural stability and textural properties, such as exceptionally large surface area, pore volume and tuneable pore sizes, provide greater drug loading and surface functionalization [30,57,58]. The great flexibility in surface functionality is owned by the silanol groups (-Si-OH) available for modification [30]. All this provides superior cargo loading capacities [58]. Additionally, HMSNs have a higher drug storage volume than MSNs due to the hollow centre of the nanoparticle [59]. 

Mesoporous materials are defined as having a pore size in the range of 2–50 nm [60]. However, typical pore diameters are between 2 and 5 nm, with different particle sizes ranging from nanoscale to microscale, with large surface area (from 700 to 1000 m^2^/g) and high pore volume (from 0.6 to 1 cm^3^/g) [61]. The pore size could be varied and tuned through the choice of surfactants used to synthesize MSNs to achieve greater loading capacity for molecules of different sizes and shapes [60]. The characteristics of the nanocarriers, such as size, shape, and pore size, are fundamental since they directly impact drug delivery. Pore size influences the loading and release of the drug. For instance, an enlarged pore might be advantageous for allocating bulky molecules such as insulin. Cellular interaction, for example, might be affected by the shape of the nanoparticles [62]. The size and surface charge of the nanoparticles can even have an impact on intestinal permeation, in the case of insulin being favoured by negatively charged small-size nanoparticles (less than 100 nm) [24]. 

**Figure 3:**
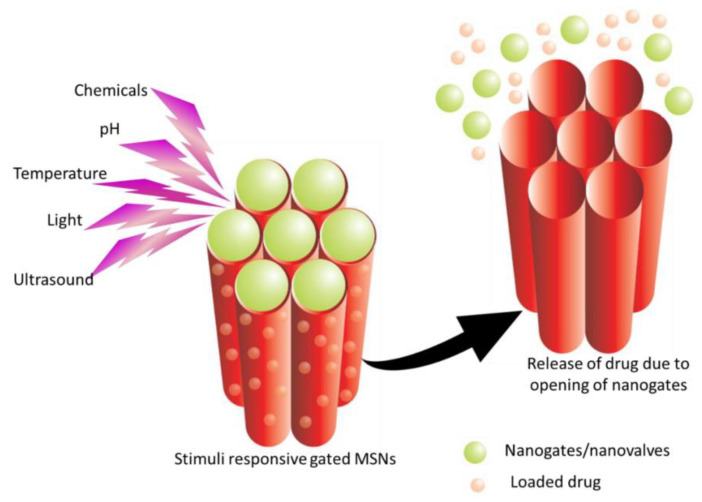
Schematic illustration of the release of a drug from gated MSNs in response to stimuli [60].

Although the morphological and textural properties of MSNs may affect the release profile, these can in principle be adjusted by appropriate surface modification. A common approach is gated release, consisting in blocking the exit of the pores after entrapping the drug inside. The release of the encapsulated molecules is triggered by endogenous (e.g., pH, redox) or exogenous (e.g., light, ultrasound) stimuli. Gatekeepers are molecules or particles that open the pores in response to these stimuli and lead to cargo release (Figure 3) [60]. In MSNs for oral delivery, pH is the most common stimulus used [13,14,15], while for other administration routes, a glucose-responsive release is the most employed [53,63,64]. 

### 3.2. Main Preparation Methods

The synthesis of MSNs is based on the Stöber method, which was developed in 1968 and still continues to be the most extensively used method to prepare non-porous silica [65]. Briefly, the Stöber method uses the sol-gel process and consists in the hydrolysis and condensation of a silica precursor (typically tetraethyl orthosilicate (TEOS), but other silicates such as tetramethyl orthosilicate (TMOS) and tetramethoxyvinylsilane (TMVS) can be used), in water and ethanol, with ammonia as catalyst. Synthesis can be achieved in acid and neutral conditions as well. The alkoxide monomers are hydrolysed and condensed into a colloidal solution (sol), which is a precursor for forming a network of polymer or discrete particles (gel). By varying the concentration of the silicate and the amount of solvent and catalyst, fine-tuning of particle size and shape is achieved [60,65]. 

**Figure 4:**
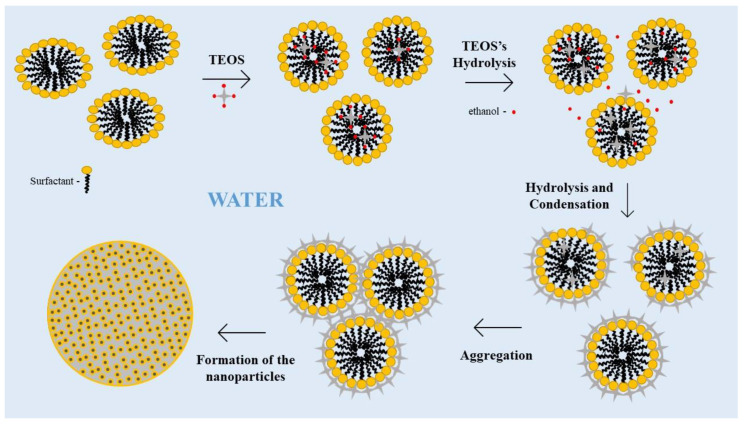
Schematic illustration of the swelling-shrinking mechanism.

The sol-gel method described above is the basis for the synthesis of MSNs. The inorganic network is formed around a soft (e.g., surfactant, polymer) or hard template (e.g., polymer beads, metal, metal oxide). The cationic surfactant-templating method using cetyl-trimethylammonium bromide (CTAB) or similar, is still the most widely used method [66]. The cationic surfactant forms ellipsoidal micelles with a hydrophobic core where TEOS becomes solubilized, thus enlarging the micelles that switch from ellipsoidal to spherical shape. The hydrolysed monomers of TEOS become hydrophilic, leaving the core of the micelle to the aqueous surroundings. Because these monomers have a negative charge density, they tend to adsorb onto the positively charged CTAB micelles via electrostatic interaction. As TEOS is consumed, the micelles become smaller until all TEOS is hydrolysed, and the silica network is formed around the micelles. Finally, the micelles aggregate, resulting in particle growth and forming a mesoporous structure [67]. The method is often referred to as swelling-shrinking mechanism (Figure 4). Non-ionic surfactants and block polymers can also be employed to prevent the irreversible aggregation of MSNs during synthesis [68] and to help control the mesostructure [69]. By varying the type and concentration of silica precursor and surfactant and reaction conditions, it is possible to synthesize mesostructured silica materials with highly tuneable properties and morphologies, including parallel hexagonal mesochannels, radially oriented mesopores and hierarchical mesostructures; for example, MCM-41 and SBA-15, which have been widely employed for drug delivery [60,67,70]. After synthesis, removal of the surfactant is necessary. This can be done by extraction or calcination, although dry air calcination is less recommended since it promotes agglomeration [66]. 

Hollow mesoporous nanoparticles (HMSNs) are prepared following similar procedures using a core template that, in the end, is removed, leaving an empty core [71]. Examples of hard templates include polymers, silica or carbon-based templates [54,72]. Soft templates take advantage of different techniques, including emulsion, micelle, vesicle-based, gas bubble and electrospray techniques. Another way to synthesize HMSNs is with self-templating methods that do not require a core template [73]. 

**Figure 5:**
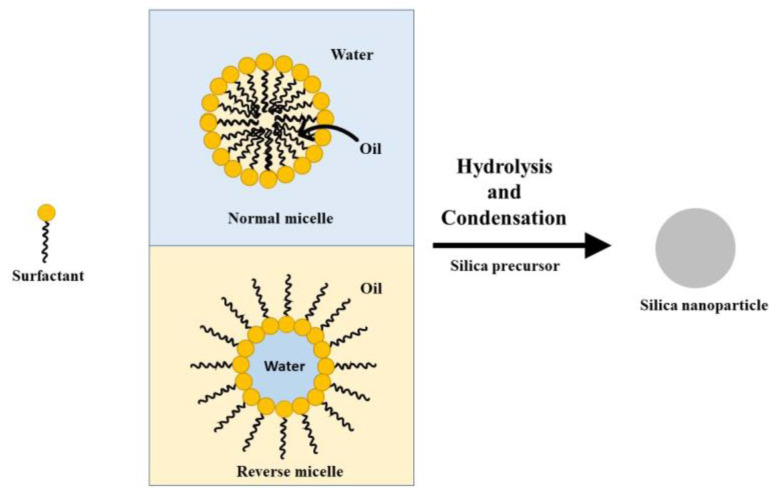
Illustration of a normal and a reverse micelle in the microemulsion method to synthesize silica nanoparticles.

Other methods have been used to synthesize silica nanoparticles. In the microemulsion method, nanodroplets of oil-in-water (o/w, normal micelle) or water-in-oil (w/o, reverse micelle) work as nanoreactors in which silica nanoparticles are formed, through hydrolysis and condensation reactions (Figure 5). The former (o/w) are suitable for synthesizing organically modified silica [74], while the latter allow for the production of ultrasmall (<10 nm) conventional amorphous silica nanoparticles [75]. Ionic or non-ionic surfactants stabilize the micelles and nanodroplets, whose diameter determines the size of the synthesized nanoparticles [76]. Microemulsions are also employed in the synthesis of hollow and yolk/shell silica nanospheres [77].

### 3.3. Cytotoxicity

Silica nanoparticles present in vitro and in vivo toxicity, as reviewed by Murugadoss et al. [78]. The in vitro toxicity of silica nanoparticles (cytotoxicity, genotoxicity and immunotoxicity) was concluded to be size-, dose- and cell type-dependent. In vivo effects (namely, endothelial dysfunction, haemolysis, and neurotoxicity) were mainly observed in acutely exposed animals and were dependent on the route of administration and physicochemical properties of silica nanoparticles. Furthermore, in vitro tests suggested that porous silica nanoparticles are less cytotoxic than non-porous silica nanoparticles [79]. Spherical MSNs are also shown to be less cytotoxic and remain longer in the bloodstream than MSNs with other shapes [80]. Despite the alleged controversy over the toxicity of silica nanoparticles [81], the use of synthetic amorphous silica as a food additive has already been approved by the Food and Drug Administration (FDA) [82] and European Food Safety Authority (EFSA) [83]. Besides, some silica nanoparticles have already been tested or approved for clinical trials [84,85,86]. MSNs were already tested in humans, in a clinical study involving 12 adult men, to enhance the bioavailability of the poorly soluble drug fenofibrate [87].

## 4. Silica-Based Nanocarriers for Glycemia Control

### 4.1. Glycemia Control Mediated by Insulin 

#### 4.1.1. Oral Insulin Delivery Nanosystems

Most therapeutic proteins are administered by the parental route. The administration of insulin for diabetes treatment is done subcutaneously. Multiple problems are associated with these invasive administration routes, which lead to poor patient compliance. Consequently, only 40% of patients achieve long-term glycaemic control. The complexity of the insulin treatment routine and the need to use needles are considered the main factors influencing low patient compliance [88]. For some patients, insulin administration implies pain, trauma and distress, especially for people with needle phobia [89]. It can also provoke infections, immune reactions, and hyperinsulinemia. Oral insulin administration could help surpass these problems, avoiding needles and mimicking a normal insulin pathway without a burst increase in insulin concentration, providing better glucose homeostasis. This would be a much more convenient way to control glucose levels, improving patients’ quality of life. Additionally, oral administration is more cost-effective because it avoids using needles and other injection materials [88]. However, the oral bioavailability of insulin is very low, since this protein faces multiple physical and chemical barriers in the gastrointestinal tract (GIT) before it can reach the bloodstream. The first barrier would be the mucous layer, which filters out drugs and proteins with a positive charge. The intestinal epithelium only allows the passive diffusion of lipophilic drugs with a molecular weight lower than 700 Da, a value far exceeded by the 5800 Da of insulin. Consequently, intestinal permeability is a critical step in the oral administration of insulin and one of the main factors responsible for the low bioavailability of the protein. Moreover, the highly acidic pH in the stomach leads to protein denaturation. Another important contribution to the low bioavailability of insulin is proteolysis through the action of enzymes such as pepsin, trypsin, chymotrypsin and elastase [89]. 

Because of all the problems associated with the low oral bioavailability of insulin, glucose level control by oral administration of insulin alone is essentially impossible. Many studies have addressed this issue by using nanomaterials, including silica nanoparticles [90]. Lamson et al. [24] have found that small silica nanoparticles (<100 nm) with a negatively charged surface act as physicochemical permeation enhancers, by binding intestinal surface receptors that mediate the opening of tight junctions. Studies with diabetic mice revealed that these nanoparticles improve the oral bioavailability of insulin when co-administrated with insulin and exenatide (further discussed in this review). In contrast to other studies, here the peptides were co-administrated with the nanoparticles and were not encapsulated. Nevertheless, this study provided the scientific community with strong evidence of the role of silica nanoparticles as intestinal permeation enhancers of peptides. This was the only study in which commercially available nanoparticles were used. 

Despite the exciting results of Lamson et al. [24], the primary approach to enhancing the oral bioavailability of insulin has been the delivery of insulin-loaded nanoparticles [13,14,15]. MSNs are very attractive for the delivery of bulky molecules such as insulin owing to their high biocompatibility, stability and loading capacity of large molecules, especially in nanoparticles with enlarged pores [91,92]. Additionally, they offer protection against multiple external agents that affect protein integrity [93]. Table 1 lists several MSN-based carriers for insulin delivery. As seen in Table 1, MSNs often need surface modification in order to modulate encapsulation, release and targeted delivery. Overall, these systems are designed to release insulin triggered by variations in pH and/or glucose. Glucose sensitivity is usually provided by functionalizing with phenylboronic acid or derivatives [94,95]. The 1,2-, 1,3- or 1,4-diols on the saccharides react with hydrophobic boronic acids, forming hydrophilic cyclic boronic esters, in a reversible reaction [96,97]. Increasing glucose concentration shifts the equilibrium towards more hydrophilic forms that can swell or disintegrate polymer coatings or gatekeepers and trigger the release of encapsulated insulin. pH sensitivity is usually achieved by surface modification with polymers containing ionizable pendant groups, i.e., with pH-sensitive polymers [98].

Jain et al. [52] developed MSN functionalized with boronic acid and coated with polyacrylic acid, a pH-sensitive polymer. The system was capable of performing glucose-sensitive and pH-dependent insulin release, i.e., it was responsive to two stimuli. The small-pore MSNs showed a fast insulin release, with approximately 80% release after 40 min at nearly neutral pH. In contrast, at simulated gastric conditions (pH = 2), the release of insulin was considered neglectable (the peptide was kept inside the pores, safe from the harsh gastric condition). This was possible because the polyacrylic acid coating contracts at such low pH conditions, imprisoning insulin inside the mesoporous. This study clearly shows the importance of polymeric coating in this type of nanoparticle, preventing unwanted early release. However, contrary to the authors, we consider that 80% release after 40 min (at intestinal and physiologic conditions) is not a sustained release. It would have been important to have control with non-coated nanoparticles in order to evaluate the role of polyacrylic acid in preventing a burst release under more neutral pH conditions. Glucose-responsive release was indeed observed, but the difference in insulin release between the lowest (10 mM) and the highest (50 mM) glucose concentration was less than 7%, which could possibly not be enough to have a significant impact on glycaemic control. Unfortunately, no data related to the possible antidiabetic effect of the system were reported, nor those related to the cellular permeation of the released insulin or the nanoparticle. The nanoparticles showed good biocompatibility. However, it was unclear if the proposed nanosystems indeed have the potential to deliver insulin, resulting in sufficient hypoglycaemic control. 

More recently, dendritic MSNs, i.e., dendrimer-like MSNs tableted with the succinylated β-lactoglobulin, were used for a pH-dependent release of insulin and protection from degradation [13]. Dendritic MSNs of various textural properties were obtained by changing the nature of the organic solvent. More specifically, MSNs prepared with aromatic solvent (toluene) presented larger pore size, volume, and surface area than MSNs prepared using cyclic (cyclohexane) or linear (hexane) organic solvents. Dendritic MSNs with pore sizes of 7.6 nm and 11.7 nm, synthesized in hexane and toluene, respectively, were subsequently tested for insulin loading and release. The loading technique, which took advantage of the opposite charges of insulin (positive) and dendritic MSNs (negative) at pH 4, led to 20 wt% loading. Tablets with succinylated β-lactoglobulin protein were prepared to endow pH-dependent release properties. This method provided a good release control, with less than 10% release in gastric fluid conditions and up to 80% release at pH 7.4. Thiol-functionalized dendritic MSNs were tested, since this functional group provides mucoadhesive properties that influence mucous permeability. However, it was found to cause protein damage, which might lead to insulin misfunction. Nevertheless, non-functionalized dendritic MSNs alone could enhance insulin permeation, suggesting that the nanocarrier as prepared could ensure intestinal cellular transport of insulin. These conclusions were supported by human epithelial colon cells’ (HCECs) live cell imaging data. These were very interesting results, showing how important permeation tests are and how the nanoparticle’s structure and functionalities can influence insulin stability and integrity. Nevertheless, the study lacked antidiabetic or glycaemic regulation tests, hindering conclusions about the real therapeutic effect. 

Other nanosystems have been reported for glycaemic regulation. For example, dendritic MSNs coated with alginate-g-3-aminophenylboronic acid and others coated with chitosan-g-3-fluoro-4-carboxyphenylboronic acid. These two nanocarriers with opposite charges, loaded with insulin, form polyelectrolyte complexes that were tested for their capacity to release insulin in response to glucose concentration. Gastric conditions promote electrostatic interactions between the two coating compounds, making it difficult for insulin to leak out of the nanoparticles, thus preventing its release. These polyelectrolyte complexes seem to better prevent insulin release at gastric conditions than the system previously discussed, with the release being less than 10% in the first 2 h. For physiological conditions (pH = 7.4), insulin release was higher when glucose concentration was higher. Although permeability studies are important to infer the bioavailability of insulin, the in vivo studies on diabetic rats demonstrated a significant hypoglycaemic effect of the system, presumably by self-regulating insulin release and enhancing the bioavailability of insulin [99]. 

Esmaeili et al. [14] used MCM-41 MSNs coated with polyamide amine dendrimer and placed in chitosan-gelatine scaffolds to deliver insulin in diabetic-induced male rats. The in vitro release was investigated in a pH range from 4.4 to 8.4 and it was observed that insulin release is retarded with increasing pH. Under physiological conditions, insulin release started after around 8 h. Such a slow release might limit the therapeutic effect. On the other hand, for lower pH values, release is much faster. At pH 4.4, 10% insulin release was observed after 2 h, which limits oral delivery due to early release in the GIT. It is essential to mention that in all pH conditions, 100% release was achieved. This means that insulin does not remain trapped inside the mesopores, showing how effective these nanoparticles are in releasing its cargo. When tested in vivo, the system did show the ability to reduce blood glucose levels in diabetic rats compared to the control. Confocal images in the liver and blood detected permeability to delivered insulin. However, glucose levels do not seem to decrease in what would be an ideal scenario. Indeed, average blood glucose values before insulin administration (between 430–440 mg/dL) dropped only to values slightly above 350 mg/dL. Despite the positive results, the work lacks some important data, such as the release profile in gastric conditions, which is vital for oral delivery of drugs, and a more detailed follow-up of glycaemic control. It would also be valuable to compare the results with a group treated with insulin administered through the traditional route. Moreover, in order to consider the applicability of oral insulin by using this carrier, more effective glycaemic control must be shown. 

**Figure 6:**
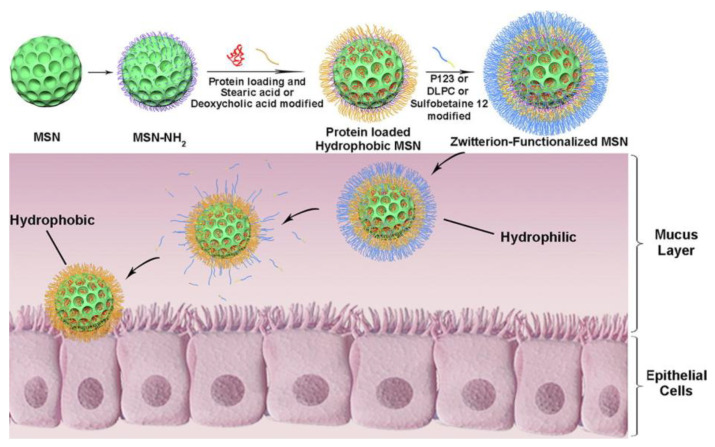
Representation of how zwitterion-functionalized MSNs were built and how they work [15]. (Reprinted with permission from [15]. Copyright Elsevier, 2021. More details on “Copyright and Licensing” are available via the following link: https://www.mdpi.com/ethics#10).

Gao et al. [15] provided a complete study using zwitterion-functionalized MSNs. Amine-functionalized MSNs were covalently linked to deoxycholic acid, a bile acid, and then coated with the zwitterion sulfobetaine [12]. The nanoparticles were hydrophobic after modification with deoxycholic acid and hydrophilic after zwitterion functionalization. This approach improved nanoparticle mucus penetrating ability and transepithelial absorption and provided remarkable affinity with epithelial cells (Figure 6). In fact, the cellular uptake of functionalized MSNs was improved by 10 and 8 times for Caco-2 and E12 cells, respectively. The results also indicated that the nanocarrier avoided lysosomes, in which harsh conditions would damage insulin. It also increased the absorption of loaded insulin in all intestinal segments and showed a hypoglycaemic effect in diabetic rats. The results showed that the blood glucose level reduced to 45% 1 h after administration and reduced to the same level as subcutaneous insulin administration after 6 h, with the hypoglycaemic effect lasting 10 h. This prolonged hypoglycaemic effect might be related to the gradual release of insulin from the nanocarrier, the high insulin loading and the stability of the loaded insulin against protease hydrolysis provided by the porous structure of MSN. This study brought very important knowledge and showed that oral delivery of insulin is possible and comparable to subcutaneously administered insulin. Zwitterion functionalization was very effective and clearly showed the important role of coating and functionalization in improving nanosystem features. It would be relevant to know the stability and integrity of insulin and the release profile in gastric conditions to better understand the route of insulin throughout the GIT into the bloodstream. This knowledge is especially important considering that other previously mentioned studies reported interactions between coating molecules and insulin that might influence its action. 

Zhang et al. [91] proposed a very exciting approach in which MSNs loaded with insulin were modified with functional groups to mimic a virus surface capable of penetrating through the mucus layer and the intestinal epithelium. The nanosystem was able to reduce blood glucose levels in diabetic rats by approximately 50%, and this effect lasted longer than that of subcutaneously administered insulin. The structural stability of insulin after being released from the coated MSNs was not significantly altered, thus maintaining its activity. Additionally, no significant toxicity was detected in preliminary in vitro or in vivo studies.

#### 4.1.2. Non-Oral Insulin Delivery Systems

Other approaches have been proposed that do not involve the oral administration of insulin, but mainly focus on the controlled release of insulin and stimuli-triggered release. As expected, diabetes treatment focused on glucose-responsive release is a common approach, allowing for a self-regulated release of insulin to avoid burst release and its consequences, such as hypoglycaemia. As mentioned before, functionalization with phenylboronic acids is a typical strategy to endow glucose-sensitive properties to the nanocarriers. In this context, Hou et al. [63], prepared MSN (average size ~190 nm) modified with carboxyphenylboronic acid. The insulin was loaded in the pores and the MSNs were coated with sodium alginate that worked as a gatekeeper for insulin. The nanosystem was intravenously administrated to diabetic mice and was able to release insulin selectively in response to glucose concentrations. With only one dose of the nanosystem, the blood glucose level was kept normal for 12 h. While this therapeutic strategy does not avoid many of the problems previously mentioned in the conventional administration route, primarily associated with needle injection, it does help prevent hyperinsulinemia, since insulin is slowly released in response to glucose concentrations.

**Figure 7:**
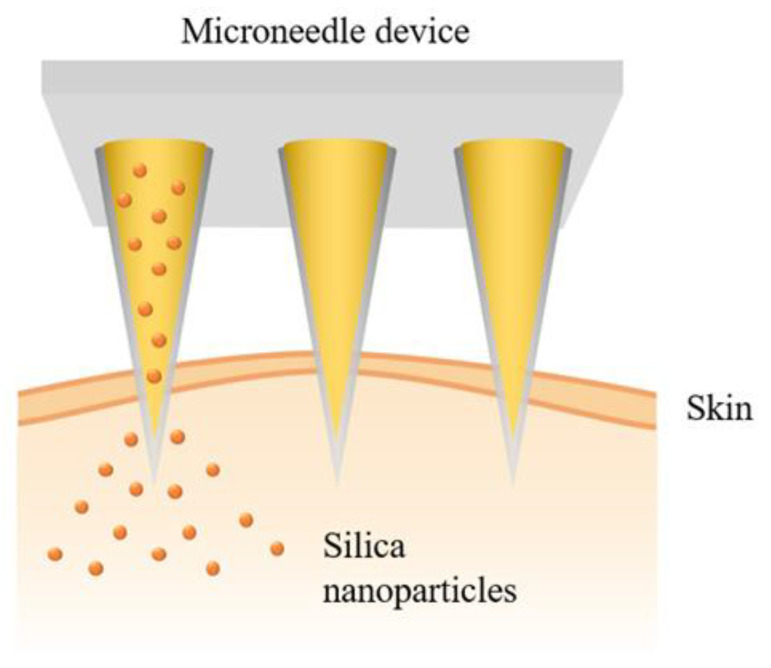
Representation of a microneedle device/patch releasing silica nanoparticles.

MSNs (100 to 120 nm) functionalized with phenylboronic acid as a glucose-responsive linker and ZnO nanoparticles (5 to 10 nm) as gatekeepers were used for controlled insulin release [53]. Here, the anthracene-based monoboric acid 3 was a sensitive linker to regulate insulin release, which was entrapped in the mesopores using a gatekeeper mechanism with zinc oxide dots that physically blocked the pores, preventing insulin from being released. The nanoparticles were administered intravenously and transdermally through a hyaluronic acid-based microneedle patch with efficient skin penetration (Figure 7). The system showed no hypoglycaemic risk in either administration way and effectively controlled blood glucose levels in type 1 diabetic rats. Additionally, the microneedle patch reduced the pain of injection and demonstrated its convenience as an administration route. Xu et al. [64] reported a similar approach, in which a microneedle device was used for transdermal administration of H_2_O_2_-responsive MSNs for the controlled release of insulin in response to glucose concentrations. Glucose oxidase was also encapsulated. This enzyme transforms glucose into gluconic acid, generating H_2_O_2_ that breaks the host–guest complexation between 4-(imidazoyl carbamate)phenylboronic acid pinacol ester and α-cyclodextrin, which is responsible for insulin confinement inside the pore, resulting in insulin release. The system was able to control blood glucose levels in diabetic rats without a hypoglycaemic effect and maintained lower blood glucose levels for longer than free insulin. In both cases, insulin release was relatively low. In the former study [53], even for the highest glucose concentration (900 mg/dL), insulin release did not reach 50%; in the latter [64], insulin release was around 70% for a higher glucose concentration (360 mg/dL). The administration route was very convenient and provided good results. However, other studies have reported MSNs with better insulin release profiles, such as the one described hereafter. 

Oroval et al. [100] synthesized enlarged pore MSNs functionalized with 1-propyl-1-H-benzimidazole and coated with cyclodextrin-modified glucose oxidase. The expanded pores, with an average pore diameter of 11.8 nm, served as an insulin storage compartment; the release of insulin was self-regulated and dependent on glucose concentration. The enzymatic reaction between glucose and the cyclodextrin-modified glucose oxidase, which acted as a gatekeeper, resulted in the hydrolysis of glucose into gluconic acid, causing a local pH drop that led to the protonation of benzimidazole groups, and consequently insulin release. In vitro tests in simulated blood plasma showed a good glucose-responsive insulin release of the nanosystem. Insulin release was dependent on glucose concentrations, achieving ca. 90% for 15 mM glucose (270 mg/dL) and reaching 100% at 40 mM (720 mg/dL). The authors predicted that the material could release the amount of insulin necessary to decrease blood glucose levels to normal values. A very important advantage of an enzymatic reaction is the high selectivity, which ensures that insulin, or any other loaded molecule, is only released when glucose is present. Despite the auspicious results of this study, no in vivo studies have been reported, limiting the conclusions about the real antidiabetic effect. 

**Figure 8:**
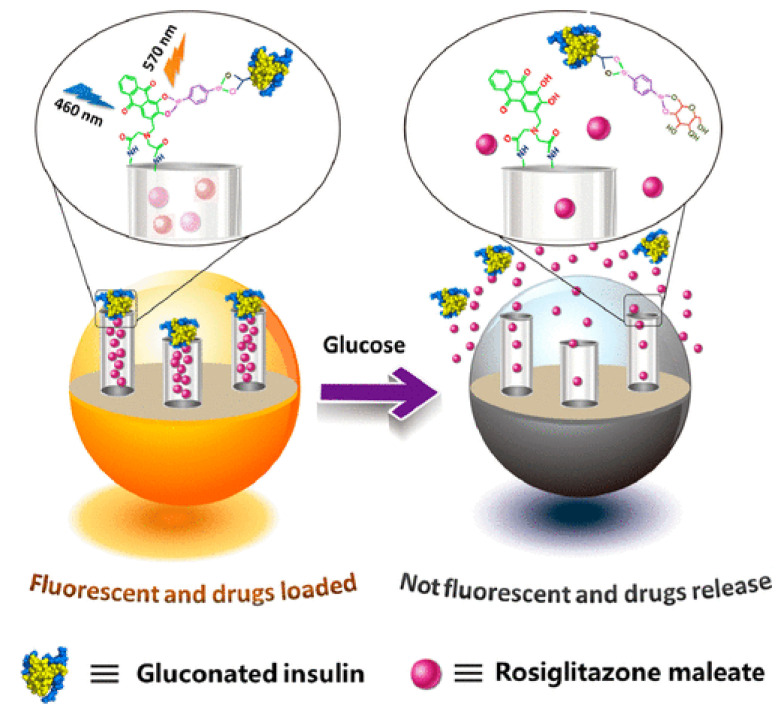
MSNs performing glucose-dependent drug release with real-time monitoring through fluorescent emission [101]. (Reprinted with permission from [101]. Copyright American Chemical Society, 2016. More details on “Copyright and Licensing” are available via the following link: https://www.mdpi.com/ethics#10).

In recent years, several works have been developed aiming to create an intelligent delivery system combining two key features, self-regulated drug release and real-time release monitoring, using different types of nanoparticles and drugs. This technology has been most studied using antitumoral drugs with various stimuli responses and monitoring techniques, including in vivo and in situ release monitoring [102,103,104,105,106]. Some successful examples of nanosystems using this approach use silica nanoparticles [107,108,109]. In the field of diabetes treatment, using smart silica nanoparticles with real-time release monitoring, less work has been done compared to antitumour drugs. In fact, we could only find one work reporting the synthesis of MSNs with both self-regulated and monitored release of an antidiabetic drug (insulin) [101]. The nanocarrier comprised amino-functionalized MSNs modified with alizarin complexone, where gluconated insulin was graphed via a benzene-1,4-diboronic acid-mediated esterification reaction. Gluconated insulin worked as a gatekeeper molecule and hypoglycaemic agent (Figure 8). When excited with 460 nm radiation, the boronated ester exhibited an emission peak at 570 nm, which can be quantified. Glucose caused the dissociation of boronate ester, which stopped fluorescent emission and set free gluconated insulin, which could perform its hypoglycaemic effect. It also led to cargo release from the mesopores, which in this case is the hypoglycaemic drug rosiglitazone maleate. The study successfully correlated loss in fluorescence emission with the release of both drugs caused by glucose concentration. Unfortunately, no tests were performed to evaluate the antidiabetic effect of the nanosystem. However, taking into consideration other studies using MSNs loaded with different hypoglycaemic drugs that had demonstrated antidiabetic effects, as well as a good release profile achieving almost 80% release for a glucose concentration of 80 mM, it seems reasonable to predict the good antidiabetic effect of this nanosystems or any other using this nanocarrier load with a different hypoglycaemic drug. 

In conclusion, previous studies have shown that mesopores have an important role in protecting insulin from external adverse conditions and provide a controlled and slow release of insulin as well, which is crucial for proper glycaemic control, and that silica nanoparticles have an essential role in promoting insulin transport across the intestinal epithelium. Furthermore, the monitoring capacity of the nanocarriers opens a whole new sight for glucose level control and diagnosis. It was clear that the versatility of silica nanoparticles regarding surface modification and functionalization and the role of the molecules used as coating agents, gatekeepers and permeability enhancers are crucial for the successful applicability of these nanosystems in therapeutic strategies. All the nanosystems mentioned above are summarized in Table 1.

### 4.2. Glycemia Control Mediated by Other Drugs

The works discussed in this section refer to the use of silica nanoparticles as nanocarriers for antidiabetic drugs other than insulin for type 2 diabetes treatment. These works are summarized in Table 2.

#### 4.2.1. Metformin Delivery Systems 

Metformin is an antidiabetic drug used in type 2 diabetes [114] and the most prescribed drug for type 2 diabetes treatment [115]. Metformin glucose-lowering effect is somewhat complex but shortly occurs by inhibiting hepatic gluconeogenesis and opposing the action of glucagon [116]. Although metformin does not have the same bioavailability problems as insulin, nanoformulations have been prepared to improve applicability. This happens, for example, by using HMSNs functionalized with poly(3-acrylamidophenylboronic acid) as a gatekeeper for metformin, which is sensitive to glucose concentration. The nanoparticles were applied in a microneedle’s device for transcutaneous application. This resulted in an intelligent glucose-responsive device that was able to control blood glucose levels with a similar performance to injected metformin. The release profile of the drug shows that regardless of the glucose concentration, metformin release stops after approximately 5 h, and for higher glucose concentration (400 mg/dL) metformin release stopped at 70%. We hypothesize that with a more prolonged and higher release of metformin (as in other previously discussed studies), glycaemic control could last longer and have a better performance than free metformin [54]. 

Silica nanoparticles can also prevent excessive drug intake. The confinement of metformin inside the pores of MSNs can cause a controlled release of the drug, thus preventing the burst concentration increase verified with isolated drug ingestion. With this purpose, Patiño-Herrera et al. [110] developed a pH-responsive system comprising MSNs loaded with metformin and coated with chitosan, to avoid excessive drug dosage. The nanosystem was able to control the release of metformin depending on the pH of the media, which in theory should reduce the amount of metformin necessary to have a therapeutic effect. However, no in vivo tests have been reported proving this hypothesis.

#### 4.2.2. Exenatide Delivery Systems

Exenatide is an analogue of glucagon-like peptide-1 (GLP-1), an important regulator of metabolic homeostasis released by intestinal L cells in response to glucose and other ingested nutrients [92]. The drug is usually given to patients with type 2 diabetes as an adjunctive therapy in cases where other drugs like metformin do not result in adequate glycaemic control. It is administrated by subcutaneous route, thus having all the related problems already mentioned for insulin. Additionally, it has a very short half-life of only 2.4 h and a bioavailability between 65 and 75%, requiring two daily doses [117]. Silica nanoparticles could help provide controlled release, extend the half-life, and enhance the bioavailability of the peptide. 

In this context, Chen et al. [111] demonstrated that the half-life time of exenatide could be improved, confining the peptide in the mesopores of MSNs, particularly in the case of rod-shaped SBA-15. The loaded MSNs were tested in vivo in diabetic mice by intravenous administration, and it was observed that the half-life time was extended up to 14.5 h and the bioavailability was increased, resulting in a prolonged hypoglycaemic effect. 

Improving the oral bioavailability of exenatide is a major challenge, but, for the same reasons as insulin, would be a crucial step towards formulating an oral therapeutic solution. This was what Abeer et al. [92] attempted to do using large-pore (~10 nm) dendritic MSNs functionalized with phosphonate groups and coated with chitosan. The in vitro permeation tests showed that bioavailability increased 1.7 times compared to unloaded exenatide. Chitosan slowed exenatide release, which is important to prevent burst release. The in vitro triple co-culture model also suggested that coating with chitosan improved the permeation of exenatide. Unfortunately, no antidiabetic, blood glucose control, or any in vivo data were reported, limiting the discussion and conclusions that could be drawn from this work. 

In summary, although only two studies involving encapsulation of exenatide in silica nanoparticles were reported, they provided interesting results that trigger curiosity for more studies involving similar strategies, with more comprehensive approaches, and more well sustained results. It would be important to provide the scientific community with data related to the stability, integrity and activity of the peptide, and more studies regarding the intestinal permeation of exenatide and its mechanisms. Also needed are studies on different nanosystems with different features for an even better release control and in vivo tests for a solid understanding of the potential of the nanosystems involving exenatide and silica nanoparticles.

#### 4.2.3. Other Delivery Systems

Glimepiride is a drug applied in the treatment of type 2 diabetes. This molecule has poor water solubility and a short half-life time of approximately 5 h. Yu et al. [72], loaded glimepiride onto HMSNs coated with gelatine to improve glimepiride water solubility and regulate its release rate. This resulted in an improved bioavailability of the drug and a more controlled release that extended the concentration of the drug for a longer period. The in vivo tests with diabetic mice showed an improved hypoglycaemic effect. 

16-Hydroxycleroda-3,13-dine-16,15-olide is a natural supplement extracted from *Polyalthia longifolia*. This natural compound showed an ability to inhibit dipeptidyl peptidase-4, which is an enzyme responsible for GLP-1 degradation, ultimately leading to increased blood glucose levels. Although 16-hydroxycleroda-3,13-dine-16,15-olide has an antidiabetic effect, its low water solubility limits its applicability. Silica nanoparticles, in this case MSNs, were used to improve the bioavailability of this compound by modulating its solubility through encapsulation. The in vivo tests in diabetic mice also revealed the potential to regulate blood glucose levels [112]. 

Liraglutide, a GLP-1 receptor agonist, has been widely used to treat type 2 diabetes. Moreover, it has been shown that fibroblast growth factor 21 improves glucose metabolism and insulin resistance. Geng et al. [113] hypothesized that these two compounds might have a synergistic effect and therefore developed MSNs functionalized with amino groups and loaded with liraglutide and a fibroblast growth factor 21 plasmid. The nanosystem lowered blood glucose levels, significantly enhanced glucose tolerance, and exhibited a synergistic effect.

Being a nanocarrier for antidiabetic drugs, silica nanoparticles have been used not only directly in regulating glucose levels and diabetes therapy but also as glucose sensors. A key step for diabetes diagnosis and control is glucose detection and quantification; however, few studies have used silica nanoparticles for that purpose. Examples are non-porous and porous silica nanoparticles in needle-type amperometric subcutaneous glucose sensors using nitric oxide [118]. Core-shell silica nanoparticles have also been used to determine H_2_O_2_ and glucose via etching silver nanoprisms [119,120]. These nanoparticles have been employed in many different studies with some relation to diabetes, including the controlled release of nutrients [121] and nitric oxide [122] in transplanted tissue. In addition, poor wound healing is a critical side effect of diabetes, for which a small number of works using silica nanoparticles have been published [123,124,125]. Other side effects of diabetes have also been studied with approaches using silica nanoparticles, including encapsulated *Echinacea purpurea* ethanol extract [126], and triterpenoids from Petri dish-cultured *Antrodia cinnamomea* [127] for modulation of diabetes-induced reproductive dysfunction, cerium(III) chloride-loaded MSNs for alleviation of diabetic cataract development and progression [128], and resveratrol-loaded MSNs in immunoregulation and insulin resistance alleviation for diabetic periodontitis therapy [129].

## 5. Conclusions and Future Perspectives

Several studies have been published in the last few years taking advantage of the outstanding characteristics of silica nanoparticles applied to diabetes treatment (more specifically, to blood glucose control and regulation). Silica’s contributions are diverse, ranging from its almost exclusive use as a reservoir, due to its mesopores with large and tuneable volume and diameter, to more complex roles, such as enhancing intestinal permeation. The applicability of silica nanoparticles is very diverse and versatile, allowing for different administration routes and modes of action. The results discussed in this review are auspicious and clearly show that silica nanoparticles can be helpful in addressing the current challenges in diabetes therapy, protecting and enhancing the bioavailability of insulin, or enhancing the solubility of poorly soluble drugs. We consider that MSNs are still little used for encapsulating antidiabetic drugs other than insulin, with only 7 studies found, and only 2 for metformin and exenatide. Considering the recent and increasing interest in phenolic compounds as antidiabetic agents and the improved therapeutic effect of polyphenols encapsulated in silica nanoparticles, we believe that this would be an important contribution in the field of antidiabetic therapy. However, more work needs to be done to address how nanoparticles and drugs interact, the real therapeutic effect and the mechanisms involved in the intestinal permeation of drugs and nanocarriers. It seems very important to deeply study MSNs as enhancers of intestinal permeation to ascertain if they have the same effect as non-porous silica nanoparticles. Transdermal drug delivery is very convenient and provides good glycaemic control. However, insulin release was not particularly high. Future works should address this topic and work on developing a nanosystem with enhanced insulin release for better glycaemic control. Although the dual functionality nanocarrier technology is still in a very early stage, the future of silica nanoparticles for diabetes treatment might be the combination of different strategies. For instance, using MSNs as glucose sensing agents and reservoirs for insulin, storing the protein for a controlled release mediated by glucose and/or pH stimuli. Lastly, it would be extremely valuable to estimate the cost-effectiveness of a potential silica nanosystem for diabetes treatment, compared to traditional alternatives, regarding production and storage and therapeutic effectiveness.

## Figures and Tables

**Table 1:** Silica nanosystems used for insulin delivery, administration route, main characteristics, and conclusions.

Administration Routes	Nanoparticle Type	Stimuli Response	Chemical Modifications	In Vivo Tested	Main Conclusions	Ref.
Oral	Non-porous silica	None	Non-modified, COO^−^ and NH_3_^+^	Yes	Enhanced intestinal permeation of insulin by negatively charged and small-size NPs	[24]
	MSNs	pH and glucose	Functionalized with boric acid and coated with polyacrylic acid	No	pH-dependent and glucose-triggered release of insulin	[52]
	Dendritic MSN	pH	Thiol groups	No	Succinylated β-lactoglobulin tablets with dendritic MSNs with good pH-dependent release (80% at pH 7.4)	[13]
	Dendritic MSN	pH and glucose	Alginate-g-3-aminophenylboronic acid or chitosan-g-3-fluoro-4-carboxyphenylboronic acid coating	Yes	Self-regulates insulin release, demonstrating a significant hypoglycaemic effect on diabetic rats	[99]
	MSNs	pH	Polyamide amine coating	Yes	Chitosan-gelatine scaffolds containing NPs enhanced permeability of insulin and reduced blood glucose levels in rats	[14]
	MSNs	pH	Deoxycholic acid and coated with sulfobeataine 12	Yes	Increased absorption of loaded insulin and hypoglycaemic effect in diabetic rats	[15]
	MSNs	None	Several virus-mimicking functional groups	Yes	Enhanced penetration through the mucus layer and epithelium and effective hypoglycaemic effect	[91]
Intravenous	MSNs	Glucose	Carboxyphenylboronic modified and sodium alginate coated	Yes	Effective blood glucose levels control in response to glucose concentration	[63]
Intravenous and transdermal	MSNs	Glucose	Phenylboronic acid zinc oxide NPs	Yes	No hypoglycaemic risk and effective blood glucose level control	[53]
Transdermal	MSNs	Glucose	4-(imidazoyl carbamate)phenylboronic acid pinacol ester, α-cyclodextrin and glucose oxidase	Yes	Blood glucose levels control without hypoglycaemic effect	[64]
Not proposed	MSNs	Glucose	1-propyl-1-H-benzimidazole and cyclodextrin-modified glucose oxidase coating	No	Self-regulated delivery system with enlarged pores with great insulin release control	[100]
	MSNs	Glucose	Alizarin complexone and gluconated insulin	No	Self-regulated release of insulin and hypoglycaemic drug rosiglitazone maleate and insulin, together with real-time release monitoring	[101]

**Table 2:** Silica nanosystems incorporating drugs for blood glucose control.

Drug	Administration Routes	Nanoparticle Type	Stimuli Response	Chemical Modifications	In Vivo Tested	Main Conclusions	Ref.
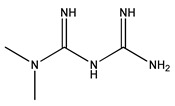 Metformin	Transdermal	HMSNs	Glucose	poly(3-acrylamidophenylboronic acid)	Yes	Effective glucose-responsive release to control blood glucose levels	[54]
	Not proposed	MSNs	pH	Chitosan coating	No	pH-dependent controlled release of metformin	[110]
Exenatide (peptide)	Intravenous	MSNs	None	None	Yes	Peptide half-life time extended up to 14.5 h, increased bioavailability and prolonged hypoglycaemic effect	[111]
	Not proposed	Dendritic MSNs	pH	Phosphonate groups and chitosan coating	No	Bioavailability of exenatide increased 1.7 times, and controlled release dependent on the pH	[92]
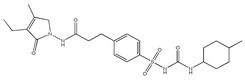 Glimepiride	Oral	HMSNs	None	Gelatine coating	Yes	Improved bioavailability of glimepiride and hypoglycaemic effect	[72]
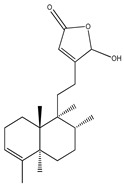 16-hydroxycleroda-3,13-dine-16,15-olide	Oral	MSNs	None	NH_2_	Yes	Improved bioavailability and blood glucose levels control	[112]
Liraglutide and fibroblast growth factor 21 (peptides)	Intravenous	MSNs	None	NH_2_	Yes	Blood glucose level control owing to the synergistic effect of both drugs	[113]

## Data Availability

Not applicable.

## References

[1] World Health Organization (2016). Global Report on Diabetes.

[2] World Health Organization (2016). Diabetes Portugal 2016 Country Profile.

[3] Hou X., Zaks T., Langer R., Dong Y. (2021). Lipid nanoparticles for mRNA delivery. Nat. Rev. Mater..

[4] Han X., Lu Y., Xie J., Zhang E., Zhu H., Du H., Wang K., Song B., Yang C., Shi Y. (2020). Zwitterionic micelles efficiently deliver oral insulin without opening tight junctions. Nat. Nanotechnol..

[5] Suzuki K., Kim K.S., Bae Y.H. (2019). Long-term oral administration of Exendin-4 to control type 2 diabetes in a rat model. J. Control. Release.

[6] Wang A., Yang T., Fan W., Yang Y., Zhu Q., Guo S., Zhu C., Yuan Y., Zhang T., Gan Y. (2019). Protein Corona Liposomes Achieve Efficient Oral Insulin Delivery by Overcoming Mucus and Epithelial Barriers. Adv. Healthc. Mater..

[7] Lee J.S., Han P., Chaudhury R., Khan S., Bickerton S., McHugh M.D., Park H.B., Siefert A.L., Rea G., Carballido J.M. (2021). Metabolic and immunomodulatory control of type 1 diabetes via orally delivered bile-acid-polymer nanocarriers of insulin or rapamycin. Nat. Biomed. Eng..

[8] Chen X., Ren Y., Feng Y., Xu X., Tan H., Li J. (2019). Cp1-11 peptide/insulin complex loaded pH-responsive nanoparticles with enhanced oral bioactivity. Int. J. Pharm..

[9] Li Y., Ji W., Peng H., Zhao R., Zhang T., Lu Z., Yang J., Liu R., Zhang X. (2021). Charge-switchable zwitterionic polycarboxybetaine particle as an intestinal permeation enhancer for efficient oral insulin delivery. Theranostics.

[10] Zhou S., Deng H., Zhang Y., Wu P., He B., Dai W., Zhang H., Zhang Q., Zhao R., Wang X. (2020). Thiolated Nanoparticles Overcome the Mucus Barrier and Epithelial Barrier for Oral Delivery of Insulin. Mol. Pharm..

[11] Zhou Y., Liu L., Cao Y., Yu S., He C., Chen X. (2020). A Nanocomposite Vehicle Based on Metal–Organic Framework Nanoparticle Incorporated Biodegradable Microspheres for Enhanced Oral Insulin Delivery. ACS Appl. Mater. Interfaces.

[12] Chen Y., Li P., Modica J.A., Drout R.J., Farha O.K. (2018). Acid-Resistant Mesoporous Metal–Organic Framework toward Oral Insulin Delivery: Protein Encapsulation, Protection, and Release. J. Am. Chem. Soc..

[13] Juère E., Caillard R., Marko D., Del Favero G., Kleitz F. (2020). Smart Protein-Based Formulation of Dendritic Mesoporous Silica Nanoparticles: Toward Oral Delivery of Insulin. Chem. Eur. J..

[14] Esmaeili A., Mousavi S.N. (2017). Synthesis of a novel structure for the oral delivery of insulin and the study of its effect on diabetic rats. Life Sci..

[15] Gao Y., He Y., Zhang H., Zhang Y., Gao T., Wang J.-H., Wang S. (2021). Zwitterion-functionalized mesoporous silica nanoparticles for enhancing oral delivery of protein drugs by overcoming multiple gastrointestinal barriers. J. Colloid Interface Sci..

[16] Li Y., Zhang W., Zhao R., Zhang X. (2022). Advances in oral peptide drug nanoparticles for diabetes mellitus treatment. Bioact. Mater..

[17] Calzoni E., Cesaretti A., Polchi A., Di Michele A., Tancini B., Emiliani C. (2019). Biocompatible Polymer Nanoparticles for Drug Delivery Applications in Cancer and Neurodegenerative Disorder Therapies. J. Funct. Biomater..

[18] Selvarajan V., Obuobi S., Ee P.L.R. (2020). Silica Nanoparticles—A Versatile Tool for the Treatment of Bacterial Infections. Front. Chem..

[19] Baek J., Robert-Nicoud G., Herrera Hidalgo C., Borg M.L., Iqbal M.N., Berlin R., Lindgren M., Waara E., Uddén A., Pietiläinen K. (2022). Engineered mesoporous silica reduces long-term blood glucose, HbA1c, and improves metabolic parameters in prediabetics. Nanomedicine.

[20] Bohara R.A., Thorat N.D., Pawar S.H. (2016). Role of functionalization: Strategies to explore potential nano-bio applications of magnetic nanoparticles. RSC Adv..

[21] Ferreira B.J.M.L., Martel F., Silva C., Santos T.M., Daniel-da-Silva A.L. (2020). Nanostructured functionalized magnetic platforms for the sustained delivery of cisplatin: Synthesis, characterization and in vitro cytotoxicity evaluation. J. Inorg. Biochem..

[22] Nogueira J., Soares S.F., Amorim C.O., Amaral J.S., Silva C., Martel F., Trindade T., Daniel-da-Silva A.L. (2020). Magnetic Driven Nanocarriers for pH-Responsive Doxorubicin Release in Cancer Therapy. Molecules.

[23] Fernandes R.A., Daniel-da-Silva A.L., Tavares A.P.M., Xavier A.M.R.B. (2017). EDTA-Cu (II) chelating magnetic nanoparticles as a support for laccase immobilization. Chem. Eng. Sci..

[24] Lamson N.G., Berger A., Fein K.C., Whitehead K.A. (2020). Anionic nanoparticles enable the oral delivery of proteins by enhancing intestinal permeability. Nat. Biomed. Eng..

[25] Aryaeian N., Khorshidi Sedehi S., Arablou T. (2017). Polyphenols and their effects on diabetes management: A review. Med. J. Islam. Repub. Iran.

[26] Shahwan M., Alhumaydhi F., Ashraf G.M., Hasan P.M.Z., Shamsi A. (2022). Role of polyphenols in combating Type 2 Diabetes and insulin resistance. Int. J. Biol. Macromol..

[27] Rocha S., Lucas M., Ribeiro D., Corvo M.L., Fernandes E., Freitas M. (2021). Nano-based drug delivery systems used as vehicles to enhance polyphenols therapeutic effect for diabetes mellitus treatment. Pharmacol. Res..

[28] Mohebian Z., Babazadeh M., Zarghami N., Mousazadeh H. (2021). Anticancer efficiency of curcumin-loaded mesoporous silica nanoparticles/nanofiber composites for potential postsurgical breast cancer treatment. J. Drug Deliv. Sci. Technol..

[29] Sarkar A., Ghosh S., Chowdhury S., Pandey B., Sil P.C. (2016). Targeted delivery of quercetin loaded mesoporous silica nanoparticles to the breast cancer cells. Biochim. Et Biophys. Acta (BBA) Gen. Subj..

[30] Chaudhary Z., Subramaniam S., Khan G.M., Abeer M.M., Qu Z., Janjua T., Kumeria T., Batra J., Popat A. (2019). Encapsulation and Controlled Release of Resveratrol Within Functionalized Mesoporous Silica Nanoparticles for Prostate Cancer Therapy. Front. Bioeng. Biotechnol..

[31] Marinheiro D., Ferreira B., Oskoei P., Oliveira H., Daniel-da-Silva A. (2021). Encapsulation and Enhanced Release of Resveratrol from Mesoporous Silica Nanoparticles for Melanoma Therapy. Materials.

[32] Ahmadi Nasab N., Hassani Kumleh H., Beygzadeh M., Teimourian S., Kazemzad M. (2018). Delivery of curcumin by a pH-responsive chitosan mesoporous silica nanoparticles for cancer treatment. Artif. Cells Nanomed. Biotechnol..

[33] Yadav Y.C., Pattnaik S., Swain K. (2019). Curcumin loaded mesoporous silica nanoparticles: Assessment of bioavailability and cardioprotective effect. Drug Dev. Ind. Pharm..

[34] (2013). American Diabetes Association Diagnosis and Classification of Diabetes Mellitus. Diabetes Care.

[35] Thevis M., Thomas A., Schänzer W., Thieme D., Hemmersbach P. (2009). Insulin. Doping in Sports.

[36] Satoh T. (2014). Molecular Mechanisms for the Regulation of Insulin-Stimulated Glucose Uptake by Small Guanosine Triphosphatases in Skeletal Muscle and Adipocytes. Int. J. Mol. Sci..

[37] (2019). AL-Ishaq; Abotaleb; Kubatka; Kajo; Büsselberg Flavonoids and Their Anti-Diabetic Effects: Cellular Mechanisms and Effects to Improve Blood Sugar Levels. Biomolecules.

[38] Sapra A., Bhandari P. (2021). Diabetes Mellitus. StatPearls.

[39] Paulweber B., Valensi P., Lindström J., Lalic N., Greaves C., McKee M., Kissimova-Skarbek K., Liatis S., Cosson E., Szendroedi J. (2010). A European Evidence-Based Guideline for the Prevention of Type 2 Diabetes. Horm. Metab. Res..

[40] (2010). The Emerging Risk Factors Collaboration Diabetes mellitus, fasting blood glucose concentration, and risk of vascular disease: A collaborative meta-analysis of 102 prospective studies. Lancet.

[41] Steinmetz J.D., Bourne R.R.A., Briant P.S., Flaxman S.R., Taylor H.R.B., Jonas J.B., Abdoli A.A., Abrha W.A., Abualhasan A., Abu-Gharbieh E.G. (2021). Causes of blindness and vision impairment in 2020 and trends over 30 years, and prevalence of avoidable blindness in relation to VISION 2020: The Right to Sight: An analysis for the Global Burden of Disease Study. Lancet Glob. Health.

[42] Saran R., Li Y., Robinson B., Ayanian J., Balkrishnan R., Bragg-Gresham J., Chen J.T.L., Cope E., Gipson D., He K. (2015). US Renal Data System 2014 Annual Data Report: Epidemiology of Kidney Disease in the United States. Am. J. Kidney Dis..

[43] Misra S., Mathieu C. (2020). Are newer insulin analogues better for people with Type 1 diabetes?. Diabet. Med..

[44] Padhi S., Nayak A.K., Behera A. (2020). Type II diabetes mellitus: A review on recent drug based therapeutics. Biomed. Pharmacother..

[45] Lorenzati B., Zucco C., Miglietta S., Lamberti F., Bruno G. (2010). Oral Hypoglycemic Drugs: Pathophysiological Basis of Their Mechanism of ActionOral Hypoglycemic Drugs: Pathophysiological Basis of Their Mechanism of Action. Pharmaceuticals.

[46] Philippe J., Raccah D. (2009). Treating type 2 diabetes: How safe are current therapeutic agents?. Int. J. Clin. Pract..

[47] Uppal S., Italiya K.S., Chitkara D., Mittal A. (2018). Nanoparticulate-based drug delivery systems for small molecule anti-diabetic drugs: An emerging paradigm for effective therapy. Acta Biomater..

[48] Souto E.B., Souto S.B., Campos J.R., Severino P., Pashirova T.N., Zakharova L.Y., Silva A.M., Durazzo A., Lucarini M., Izzo A.A. (2019). Nanoparticle Delivery Systems in the Treatment of Diabetes Complications. Molecules.

[49] Wang A.Z., Langer R., Farokhzad O.C. (2012). Nanoparticle Delivery of Cancer Drugs. Annu. Rev. Med..

[50] Díaz M.R., Vivas-Mejia P.E. (2013). Nanoparticles as drug delivery systems in cancer medicine: Emphasis on RNAi-containing nanoliposomes. Pharmaceuticals.

[51] Bamburowicz-Klimkowska M., Poplawska M., Grudzinski I.P. (2019). Nanocomposites as biomolecules delivery agents in nanomedicine. J. Nanobiotechnol..

[52] Jain R.N., Huang X., Das S., Silva R., Ivanova V., Minko T., Asefa T. (2014). Functionalized Mesoporous Silica Nanoparticles for Glucose- and pH-Stimulated Release of Insulin. Z. Anorg. Allg. Chem..

[53] Fu Y., Liu P., Chen M., Jin T., Wu H., Hei M., Wang C., Xu Y., Qian X., Zhu W. (2022). On-demand transdermal insulin delivery system for type 1 diabetes therapy with no hypoglycemia risks. J. Colloid Interface Sci..

[54] Wang Y., Cheng S., Hu W., Lin X., Cao C., Zou S., Tong Z., Jiang G., Kong X. (2021). Polymer-grafted hollow mesoporous silica nanoparticles integrated with microneedle patches for glucose-responsive drug delivery. Front. Mater. Sci..

[55] Jeelani P.G., Mulay P., Venkat R., Ramalingam C. (2020). Multifaceted Application of Silica Nanoparticles. A Review. Silicon.

[56] Tang J., Slowing I.I., Huang Y., Trewyn B.G., Hu J., Liu H., Lin V.S.Y. (2011). Poly(lactic acid)-coated mesoporous silica nanosphere for controlled release of venlafaxine. J. Colloid Interface Sci..

[57] Shen Y., Cao B., Snyder N.R., Woeppel K.M., Eles J.R., Cui X.T. (2018). ROS responsive resveratrol delivery from LDLR peptide conjugated PLA-coated mesoporous silica nanoparticles across the blood-brain barrier. J. Nanobiotechnol..

[58] Anirudhan T.S., Nair A.S. (2018). Temperature and ultrasound sensitive gatekeepers for the controlled release of chemotherapeutic drugs from mesoporous silica nanoparticles. J. Mater. Chem. B.

[59] Liu J., Luo Z., Zhang J., Luo T., Zhou J., Zhao X., Cai K. (2016). Hollow mesoporous silica nanoparticles facilitated drug delivery via cascade pH stimuli in tumor microenvironment for tumor therapy. Biomaterials.

[60] Narayan R., Nayak U.Y., Raichur A.M., Garg S. (2018). Mesoporous silica nanoparticles: A comprehensive review on synthesis and recent advances. Pharmaceutics.

[61] Maleki A., Kettiger H., Schoubben A., Rosenholm J.M., Ambrogi V., Hamidi M. (2017). Mesoporous silica materials: From physico-chemical properties to enhanced dissolution of poorly water-soluble drugs. J. Control. Release.

[62] Kankala R.K., Han Y.-H., Xia H.-Y., Wang S.-B., Chen A.-Z. (2022). Nanoarchitectured prototypes of mesoporous silica nanoparticles for innovative biomedical applications. J. Nanobiotechnol..

[63] Hou L., Zheng Y., Wang Y., Hu Y., Shi J., Liu Q., Zhang H., Zhang Z. (2018). Self-Regulated Carboxyphenylboronic Acid-Modified Mesoporous Silica Nanoparticles with “Touch Switch” Releasing Property for Insulin Delivery. ACS Appl. Mater. Interfaces.

[64] Xu B., Jiang G., Yu W., Liu D., Zhang Y., Zhou J., Sun S., Liu Y. (2017). H_2_O_2_-Responsive mesoporous silica nanoparticles integrated with microneedle patches for the glucose-monitored transdermal delivery of insulin. J. Mater. Chem. B.

[65] Stöber W., Fink A., Bohn E. (1968). Controlled growth of monodisperse silica spheres in the micron size range. J. Colloid Interface Sci..

[66] Wu S.-H., Mou C.-Y., Lin H.-P. (2013). Synthesis of mesoporous silica nanoparticles. Chem. Soc. Rev..

[67] Yi Z., Dumée L.F., Garvey C.J., Feng C., She F., Rookes J.E., Mudie S., Cahill D.M., Kong L. (2015). A New Insight into Growth Mechanism and Kinetics of Mesoporous Silica Nanoparticles by in situ Small Angle X-ray Scattering. Langmuir.

[68] Suzuki K., Ikari K., Imai H. (2004). Synthesis of Silica Nanoparticles Having a Well-Ordered Mesostructure Using a Double Surfactant System. J. Am. Chem. Soc..

[69] Peng B., Zong Y.-X., Nie M.-Z., Shan B.-Q., Yang T.-Q., Hao P., Ma S.-Y., Lam K.-F., Zhang K. (2019). Interfacial charge shielding directs the synthesis of dendritic mesoporous silica nanospheres by a dual-templating approach. N. J. Chem..

[70] Galarneau A., Cambon H., Di Renzo F., Ryoo R., Choi M., Fajula F. (2003). Microporosity and connections between pores in SBA-15 mesostructured silicas as a function of the temperature of synthesis. N. J. Chem..

[71] Sharma J., Polizos G. (2020). Hollow Silica Particles: Recent Progress and Future Perspectives. Nanomaterials.

[72] Yu X., Liu T., Lin R. (2020). Development and Characterization of a Glimepiride-Loaded Gelatin-Coated Mesoporous Hollow Silica Nanoparticle Formulation and Evaluation of Its Hypoglycemic Effect on Type-2 Diabetes Model Rats. ASSAY Drug Dev. Technol..

[73] Soares S.F., Fernandes T., Daniel-da-Silva A.L., Trindade T. (2019). The controlled synthesis of complex hollow nanostructures and prospective applications. Proc. R. Soc. A..

[74] Kim S., Ohulchanskyy T.Y., Bharali D., Chen Y., Pandey R.K., Prasad P.N. (2009). Organically Modified Silica Nanoparticles with Intraparticle Heavy-Atom Effect on the Encapsulated Photosensitizer for Enhanced Efficacy of Photodynamic Therapy. J. Phys. Chem. C.

[75] Yamauchi H., Ishikawa T., Kondo S. (1989). Surface characterization of ultramicro spherical particles of silica prepared by w/o microemulsion method. Colloids Surf..

[76] Shirshahi V., Soltani M. (2015). Solid silica nanoparticles: Applications in molecular imaging. Contrast Media Mol. Imaging.

[77] Lin Y.-S., Wu S.-H., Tseng C.-T., Hung Y., Chang C., Mou C.-Y. (2009). Synthesis of hollow silica nanospheres with a microemulsion as the template. Chem. Commun..

[78] Murugadoss S., Lison D., Godderis L., Van Den Brule S., Mast J., Brassinne F., Sebaihi N., Hoet P.H. (2017). Toxicology of silica nanoparticles: An update. Arch. Toxicol..

[79] Maurer-Jones M.A., Lin Y.-S., Haynes C.L. (2010). Functional Assessment of Metal Oxide Nanoparticle Toxicity in Immune Cells. ACS Nano.

[80] Huang X., Teng X., Chen D., Tang F., He J. (2010). The effect of the shape of mesoporous silica nanoparticles on cellular uptake and cell function. Biomaterials.

[81] Kim I.-Y., Joachim E., Choi H., Kim K. (2015). Toxicity of silica nanoparticles depends on size, dose, and cell type. Nanomed. Nanotechnol. Biol. Med..

[82] Code of Federal Regulations § 172.480 Silicon Dioxide. https://www.ecfr.gov/current/title-21/chapter-I/subchapter-B/part-172/subpart-E/section-172.480.

[83] Younes M., Aggett P., Aguilar F., Crebelli R., Dusemund B., Filipič M., Frutos M.J., Galtier P., Gott D., EFSA Panel on Food Additives and Nutrient Sources added to Food (ANS) (2018). Re-evaluation of silicon dioxide (E 551) as a food additive. EFSA J..

[84] Zanoni D.K., Stambuk H.E., Madajewski B., Montero P.H., Matsuura D., Busam K.J., Ma K., Turker M.Z., Sequeira S., Gonen M. (2021). Use of Ultrasmall Core-Shell Fluorescent Silica Nanoparticles for Image-Guided Sentinel Lymph Node Biopsy in Head and Neck Melanoma: A Nonrandomized Clinical Trial. JAMA Netw Open.

[85] Anselmo A.C., Mitragotri S. (2016). Nanoparticles in the clinic. Bioeng. Transl. Med..

[86] Janjua T.I., Cao Y., Yu C., Popat A. (2021). Clinical translation of silica nanoparticles. Nat. Rev. Mater..

[87] Bukara K., Schueller L., Rosier J., Martens M.A., Daems T., Verheyden L., Eelen S., Van Speybroeck M., Libanati C., Martens J.A. (2016). Ordered mesoporous silica to enhance the bioavailability of poorly water-soluble drugs: Proof of concept in man. Eur. J. Pharm. Biopharm..

[88] Fonte P., Araújo F., Reis S., Sarmento B. (2013). Oral Insulin Delivery: How Far are We?. J. Diabetes Sci. Technol..

[89] Gedawy A., Martinez J., Al-Salami H., Dass C.R. (2018). Oral insulin delivery: Existing barriers and current counter-strategies. J. Pharm. Pharmacol..

[90] Andreani T., de Souza A.L.R., Kiill C.P., Lorenzón E.N., Fangueiro J.F., Calpena A.C., Chaud M.V., Garcia M.L., Gremião M.P.D., Silva A.M. (2014). Preparation and characterization of PEG-coated silica nanoparticles for oral insulin delivery. Int. J. Pharm..

[91] Zhang Y., Xiong M., Ni X., Wang J., Rong H., Su Y., Yu S., Mohammad I.S., Leung S.S.Y., Hu H. (2021). Virus-Mimicking Mesoporous Silica Nanoparticles with an Electrically Neutral and Hydrophilic Surface to Improve the Oral Absorption of Insulin by Breaking Through Dual Barriers of the Mucus Layer and the Intestinal Epithelium. ACS Appl. Mater. Interfaces.

[92] Abeer M.M., Meka A.K., Pujara N., Kumeria T., Strounina E., Nunes R., Costa A., Sarmento B., Hasnain S.Z., Ross B.P. (2019). Rationally Designed Dendritic Silica Nanoparticles for Oral Delivery of Exenatide. Pharmaceutics.

[93] Carlsson N., Gustafsson H., Thörn C., Olsson L., Holmberg K., Åkerman B. (2014). Enzymes immobilized in mesoporous silica: A physical–chemical perspective. Adv. Colloid Interface Sci..

[94] Huang Q., Wang L., Yu H., Ur-Rahman K. (2019). Advances in phenylboronic acid-based closed-loop smart drug delivery system for diabetic therapy. J. Control. Release.

[95] Ma R., Shi L. (2014). Phenylboronic acid-based glucose-responsive polymeric nanoparticles: Synthesis and applications in drug delivery. Polym. Chem..

[96] Sun X., James T.D. (2015). Glucose Sensing in Supramolecular Chemistry. Chem. Rev..

[97] Brooks W.L.A., Deng C.C., Sumerlin B.S. (2018). Structure–Reactivity Relationships in Boronic Acid–Diol Complexation. ACS Omega.

[98] Shi Z., Li Q., Mei L. (2020). pH-Sensitive nanoscale materials as robust drug delivery systems for cancer therapy. Chin. Chem. Lett..

[99] Qin T., Yan L., Wang X., Lin S., Zeng Q. (2021). Glucose-Responsive Polyelectrolyte Complexes Based on Dendritic Mesoporous Silica for Oral Insulin Delivery. AAPS PharmSciTech.

[100] Oroval M., Díez P., Aznar E., Coll C., Marcos M.D., Sancenón F., Villalonga R., Martínez-Máñez R. (2017). Self-Regulated Glucose-Sensitive Neoglycoenzyme-Capped Mesoporous Silica Nanoparticles for Insulin Delivery. Chem. Eur. J..

[101] Zou Z., He D., Cai L., He X., Wang K., Yang X., Li L., Li S., Su X. (2016). Alizarin Complexone Functionalized Mesoporous Silica Nanoparticles: A Smart System Integrating Glucose-Responsive Double-Drugs Release and Real-Time Monitoring Capabilities. ACS Appl. Mater. Interfaces.

[102] Weinstain R., Segal E., Satchi-Fainaro R., Shabat D. (2010). Real-time monitoring of drug release. Chem. Commun..

[103] Ock K., Jeon W.I., Ganbold E.O., Kim M., Park J., Seo J.H., Cho K., Joo S.-W., Lee S.Y. (2012). Real-Time Monitoring of Glutathione-Triggered Thiopurine Anticancer Drug Release in Live Cells Investigated by Surface-Enhanced Raman Scattering. Anal. Chem..

[104] Zhang J., Li S., An F.-F., Liu J., Jin S., Zhang J.-C., Wang P.C., Zhang X., Lee C.-S., Liang X.-J. (2015). Self-carried curcumin nanoparticles for in vitro and in vivo cancer therapy with real-time monitoring of drug release. Nanoscale.

[105] Jana A., Devi K.S.P., Maiti T.K., Singh N.D.P. (2012). Perylene-3-ylmethanol: Fluorescent Organic Nanoparticles as a Single-Component Photoresponsive Nanocarrier with Real-Time Monitoring of Anticancer Drug Release. J. Am. Chem. Soc..

[106] Yang Z., Song J., Tang W., Fan W., Dai Y., Shen Z., Lin L., Cheng S., Liu Y., Niu G. (2019). Stimuli-Responsive Nanotheranostics for Real-Time Monitoring Drug Release by Photoacoustic Imaging. Theranostics.

[107] Zhang P., Wu T., Kong J.-L. (2014). In Situ Monitoring of Intracellular Controlled Drug Release from Mesoporous Silica Nanoparticles Coated with pH-Responsive Charge-Reversal Polymer. ACS Appl. Mater. Interfaces.

[108] Lai J., Shah B.P., Zhang Y., Yang L., Lee K.-B. (2015). Real-Time Monitoring of ATP-Responsive Drug Release Using Mesoporous-Silica-Coated Multicolor Upconversion Nanoparticles. ACS Nano.

[109] Chen Y., Lu W., Guo Y., Zhu Y., Song Y. (2020). Chitosan-Gated Fluorescent Mesoporous Silica Nanocarriers for the Real-Time Monitoring of Drug Release. Langmuir.

[110] Patiño-Herrera R., Louvier-Hernández J.F., Escamilla-Silva E.M., Chaumel J., Escobedo A.G.P., Pérez E. (2019). Prolonged release of metformin by SiO2 nanoparticles pellets for type II diabetes control. Eur. J. Pharm. Sci..

[111] Chen C., Zheng H., Xu J., Shi X., Li F., Wang X. (2017). Sustained-release study on Exenatide loaded into mesoporous silica nanoparticles: In vitro characterization and in vivo evaluation. DARU J. Pharm. Sci..

[112] Huang P.-K., Lin S.-X., Tsai M.-J., Leong M., Lin S.-R., Kankala R., Lee C.-H., Weng C.-F. (2017). Encapsulation of 16-Hydroxycleroda-3,13-Dine-16,15-Olide in Mesoporous Silica Nanoparticles as a Natural Dipeptidyl Peptidase-4 Inhibitor Potentiated Hypoglycemia in Diabetic Mice. Nanomaterials.

[113] Geng S., Qin L., He Y., Li X., Yang M., Li L., Liu D., Li Y., Niu D., Yang G. (2021). Effective and safe delivery of GLP-1AR and FGF-21 plasmids using amino-functionalized dual-mesoporous silica nanoparticles in vitro and in vivo. Biomaterials.

[114] Corcoran C., Jacobs T.F. (2022). Metformin. StatPearls.

[115] Wang Y.-W., He S.-J., Feng X., Cheng J., Luo Y.-T., Tian L., Huang Q. (2017). Metformin: A review of its potential indications. Drug Des. Dev. Ther..

[116] Pernicova I., Korbonits M. (2014). Metformin—mode of action and clinical implications for diabetes and cancer. Nat. Rev. Endocrinol..

[117] Bray G.M. (2006). Exenatide. Am. J. Health-Syst. Pharm..

[118] Malone-Povolny M.J., Merricks E.P., Wimsey L.E., Nichols T.C., Schoenfisch M.H. (2019). Long-Term Accurate Continuous Glucose Biosensors via Extended Nitric Oxide Release. ACS Sens..

[119] Lu H., Yu C., Zhang Y., Xu S. (2019). Efficient core shell structured dual response ratiometric fluorescence probe for determination of H_2_O_2_ and glucose via etching of silver nanoprisms. Anal. Chim. Acta.

[120] Lu H., Yu C., Quan S., Xu S. (2019). A novel dual response ratiometric fluorescent probe for the determination of H_2_O_2_ and glucose *via* etching of silver nanoparticles. Analyst.

[121] Razavi M., Primavera R., Kevadiya B.D., Wang J., Ullah M., Buchwald P., Thakor A.S. (2022). Retraction of “Controlled Nutrient Delivery to Pancreatic Islets Using Polydopamine-Coated Mesoporous Silica Nanoparticles”. Nano Lett..

[122] Malone-Povolny M.J., Bradshaw T.M., Merricks E.P., Long C.T., Nichols T.C., Schoenfisch M.H. (2021). Combination of Nitric Oxide Release and Surface Texture for Mitigating the Foreign Body Response. ACS Biomater. Sci. Eng..

[123] Ren X., Han Y., Wang J., Jiang Y., Yi Z., Xu H., Ke Q. (2018). An aligned porous electrospun fibrous membrane with controlled drug delivery—An efficient strategy to accelerate diabetic wound healing with improved angiogenesis. Acta Biomater..

[124] López-Goerne T., Ramírez P., Alvarez D., Rodríguez-Reinoso F., Silvestre-Albero A.M., Gómez E., Rodríguez-Castellon E. (2018). Physicochemical properties and in vivo evaluation of Pt/TiO_2_ –SiO_2_ nanopowders. Nanomedicine.

[125] Gan J., Liu C., Li H., Wang S., Wang Z., Kang Z., Huang Z., Zhang J., Wang C., Lv D. (2019). Accelerated wound healing in diabetes by reprogramming the macrophages with particle-induced clustering of the mannose receptors. Biomaterials.

[126] Mao C.-F., Zhang X.-R., Johnson A., He J.-L., Kong Z.-L. (2018). Modulation of Diabetes Mellitus-Induced Male Rat Reproductive Dysfunction with Micro-Nanoencapsulated *Echinacea purpurea* Ethanol Extract. BioMed Res. Int..

[127] Sudirman S., Hsu Y.-H., Johnson A., Tsou D., Kong Z.-L. (2018). Amelioration effects of nanoencapsulated triterpenoids from petri dish-cultured Antrodia cinnamomea on reproductive function of diabetic male rats. Int. J. Nanomed..

[128] Yang J., Gong X., Fang L., Fan Q., Cai L., Qiu X., Zhang B., Chang J., Lu Y. (2017). Potential of CeCl_3_ @mSiO_2_ nanoparticles in alleviating diabetic cataract development and progression. Nanomed. Nanotechnol. Biol. Med..

[129] Tan Y., Feng J., Xiao Y., Bao C. (2022). Grafting resveratrol onto mesoporous silica nanoparticles towards efficient sustainable immunoregulation and insulin resistance alleviation for diabetic periodontitis therapy. J. Mater. Chem. B.

